# *Acer tegmentosum Maxim* Inhibits Adipogenesis in *3t3-l1* Adipocytes and Attenuates Lipid Accumulation in Obese Rats Fed a High-Fat Diet

**DOI:** 10.3390/nu12123753

**Published:** 2020-12-07

**Authors:** Hang-Hee Cho, Soo-Jung Lee, Sung-Ho Kim, Sun-Hee Jang, Chungkil Won, Hong-Duck Kim, Tae Hoon Kim, Jae-Hyeon Cho

**Affiliations:** 1 Institute of Animal Medicine, College of Veterinary Medicine, Gyeongsang National University, Jinju 660-701, Korea; greatcho2000@nate.com (H.-H.C.); insectraon@naver.com (S.-H.K.); sunhee5321@naver.com (S.-H.J.); wonck@gnu.ac.kr (C.W.); 2 Department of Foods and Nutrition, Gyeongsang National University, Jinju 660-701, Korea; bodry96@hanmail.net; 3 Department of Public Health, Division of Environmental Health Science, New York Medical College, Valhalla, NY 10595, USA; HongDuck_Kim@nymc.edu; 4 Department of Food Science and Biotechnology, Daegu University, Gyungsan 712-714, Korea

**Keywords:** *Acer tegmentosum Maxim*, high-fat diet, *3T3-L1* cells, adipocyte differentiation, obesity

## Abstract

We investigated the effect of *Acer tegmentosum Maxim* (ATM) on adipocyte differentiation in *3T3-L1* cells and anti-obesity properties in obese rats fed a high-fat diet (HFD). Cellular lipid content in DMI (dexamethasone, 3–isobutyl–1–methylxanthine, and insulin mixture)-treated cells increased, while ATM treatment caused a significant reduction in lipid accumulation in differentiated *3T3-L1* cells. ATM (60 ug/mL) caused inhibition of adipogenesis via down-regulation of the CCAAT/enhancer binding protein β (*C/EBPβ*) (48%), *C/EBPα* (66%), and peroxisome proliferator-activated receptor γ (*PPARγ*) (64%) expressions in *3T3-L1* cells. Moreover, ATM induced a decrease in the expressions of adipocyte-specific genes, such as adipocyte fatty acid-binding protein-2 (*aP2*), fatty acid synthase (*FAS*), and lipoprotein lipase (*LPL*). Protein kinase B (*Akt*) and glycogen synthase kinase 3β (*GSK3β*) phosphorylation was also decreased by ATM treatment of *3T3-L1* adipocytes. We investigated the anti-obesity effects of ATM on HFD-induced obese rats. Rats fed with an HFD demonstrated elevations in body weight gain, while the administration of ATM reversed body weight (BW) gains and adipose tissue weights in rats fed an HFD. ATM supplementation caused a decrease in the circulating triglyceride and total cholesterol levels and led to inhibition of lipid accumulation in the adipose tissues in HFD-induced obese rats. Epididymal fat exhibited significantly larger adipocytes in the HFD group than it did in the ATM-treated group. These results demonstrate that ATM administration caused a reduction in adiposity via attenuation in adipose tissue mass and adipocyte size.

## 1. Introduction

Obesity is a major risk factor for many metabolic disorders, including hyperlipidemia, type 2 diabetes, atherosclerosis, nonalcoholic fatty liver disease, and cardiovascular diseases [1]. Fat is properly defined as adipose tissue and is a biological caloric reservoir that expands in response to overnutrition and releases lipids in response to energy deficient conditions. Although adipose tissues are the major site for excess energy storage, excess adiposity and adipocyte dysfunction cause induction of increased levels of plasma free fatty acids and triglycerides (TGs), decreased levels of high-density lipoprotein (HDL), and abnormal low-density lipoprotein (LDL) composition in plasma and tissues, such as liver and muscle. These changes lead to pathological dysfunction of these tissues [2,3]. In addition, it is important for an individual to maintain an adequate balance between energy accumulation and consumption in order to prevent and improve obesity.

Adipose cell hyperplasia (cell number increase) and hypertrophy (cell size increase) might be an important factor in obesity development. Adipocyte differentiation and intracellular lipid accumulation are associated with the occurrence and development of obesity [4]. Adipogenesis is a differentiation process by which undifferentiated preadipocytes are converted to fully differentiated adipocytes. Thus, inhibition of adipogenesis and adipocyte differentiation from fibroblast-like preadipocytes into fully mature adipocytes in addition to identifying critical factors that regulate these processes is extremely important in an approach to primary obesity prevention and treatment.

The *3T3-L1* cell line is a well-established and widely used in vitro model of obesity for assessing adipocyte differentiation [5]. The differentiation process of *3T3-L1* cells into adipocytes follows a precisely ordered and temporal series of events regulated by a number of transcription factors and several signaling pathways for the development of the mature lipid-filled adipocytes [6,7]. During the differentiation process, the many key transcriptional factors that are involved, such as the CCAAT enhancer binding protein δ (C/EBPδ) and C/EBPβ, work in conjunction to induce peroxisome proliferator-activated receptor *γ* (*PPARγ*) and *C/EBPα* expressions [8]. C/EBPβ and C/EBPδ are expressed comparatively early in adipogenesis; this event is an important factor for initiating the transcriptional cascades that culminate in the expression of two essential adipogenic factors, *PPARγ* and C/EBPα [9]. *PPARγ* and C/EBPα are critical transcription factors involved in adipogenesis and regulation of the genes, such as lipoprotein lipase (LPL), adipocyte fatty acid-binding protein-2 (*aP2*), and fatty acid synthase (FAS) [10,11], which are involved in adipogenesis and lipogenesis. Moreover, these transcription factors are also known to influence adipocyte differentiation in vivo.

The protein kinase B (Akt) signaling pathway plays a major role in adipocyte differentiation induced by a downstream function of insulin, and activation of the Akt pathway in *3T3-L1* preadipocytes contributes to adipocyte differentiation [12], whereas inactivation of the *PI3K*/Akt pathway inhibits adipogenesis [13]. Akt phosphorylates and regulates a number of substrates involved in a diverse array of biological processes [14] and is essential for induction of *PPARγ* expression [15]. Moreover, overexpression of constitutively active Akt causes an increase in glucose uptake and adipocyte differentiation in *3T3-L1* adipocytes [16]. In contrast, the RNAi-mediated decrease in Akt expression blocks *3T3-L1* cell differentiation [17]. Adipocyte differentiation and lipid accumulation is impaired in the absence of Akt, whereas Akt overexpression in Akt-deficient cells leads to lipid droplet accumulation [18].

*Acer tegmentosum Maxim* (ATM) is widely used as a traditional medicinal plant in *Asia. A. tegmentosum* has traditionally been used for treatment of various hepatic disorders, including hepatitis, cirrhosis, and liver cancer [19]. Phytochemical compounds isolated from *A. tegmentosum* have been reported to be one of the important sources of useful bioactive secondary metabolites, such as flavonoids, lignans, and phenolic compounds [20,21]. In addition, antioxidants, anti-inflammatory, and anti-lipogenic activities were also shown in biological investigations on ATM extracts [22,23]. However, the anti-obesity effects of ATM are still poorly understood.

In the present study, we examined the inhibitory effects of ATM on adipogenesis, its underlying molecular mechanisms in *3T3-L1* cells, and body weight reduction effects in obese rats fed a high-fat diet (HFD).

## 2. Materials and Methods

### 2.1. Preparation of ATM

The *Acer tegmentosum Maxim* (ATM) stem was purchased from the Jinju traditional herbal market (GyeongsangNam-do, Jinju, Korea). The dried and chopped twigs and inner barks of ATM was powdered and extracted with 70% ethanol at 60 °C for 72 h. The extract was filtered, dried under reduced pressure, and concentrated by an evaporator to obtain an ethanol-soluble fraction. Lyophilized ATM powder was dissolved with dimethyl sulfoxide (DMSO, Sigma-Aldrich, St. Louis, MO, USA) at a concentration of 200 mg/mL and stored at −20 °C for further use.

### 2.2. Cell Culture

*3T3-L1* preadipocytes obtained from the Korean Cell Line Bank (Seoul, Korea) were cultured in Dulbecco’s modified eagle high-glucose medium (DMEM) supplemented with 10% calf serum at 37 °C in a humidified atmosphere of 5% CO_2_. To induce differentiation, 2-day post-confluent preadipocytes (designated day 0) were cultured and induced with desipramine (DMI) differentiation medium (0.5 mM 3–isobutyl–1–methylxanthine, 1 μM dexamethasone, and 0.5 μg/mL insulin in DMEM containing 10% FBS). The 3–isobutyl–1–methylxanthine (MIX), dexamethasone (DEX), insulin, and Oil Red O were obtained from Sigma-Aldrich (St. Louis, MO, USA). The medium was changed every 2 days. ATM extracts were added to the culture medium of the adipocytes on day 0. The cells were treated with 0, 20, or 60 µg/mL ATM extracts every day. After treatment with ATM for 4 and 8 days, the *3T3-L1* adipocytes were lysed for Western blot analysis. To analyze cell viability, the cytotoxicity of the ATM was evaluated using 3–(4,5–demethylthiazol–2–yl)-2,5–diphenyltetrazolium bromide (MTT).

### 2.3. Oil Red O Staining and Triglyceride Assay

After the induction of differentiation, cells were stained with Oil Red O (60% isopropanol and 40% water). Briefly, the cells were gently washed with phosphate-buffered saline (PBS) and stained with filtered Oil Red O solution for 30 min. After staining, the Oil Red O staining solution was removed, and the plates were rinsed with water and then dried. The stained lipid droplets were viewed on an Olympus microscope (Olympus, Tokyo, Japan). To analyze the content of the cellular triglycerides, the cells were washed with PBS, scraped into 200 μL of PBS, and sonicated for 1 min. The lysates were assayed for their total triglyceride content using assay kits from Sigma-Aldrich (St. Louis, MO, USA) and for cellular protein using the Bio-Rad protein assay (Berkeley, CA, USA). The results were expressed as mg of triglyceride per mg of cellular protein.

### 2.4. RT-PCR

Cellular RNA was extracted from *3T3-L1* adipocytes or epididymal adipocyte tissue using the Trizol reagent (Invitrogen, Carlsbad, CA, USA) according to the manufacturer’s instructions. One microgram of total RNA was used for the first strand of cDNA synthesis with oligo (deoxythymidine) primers and Superscript II reverse transcriptase (Invitrogen, CA, USA). The target cDNA was amplified using the following primers: *C/EBPβ*, 5′-GACTACGCAACACACGTGTAACT-3′ and 5′-CAAAACCAAAAACATCAACAACCC-3′; *PPARγ*, 5′-TTTTCAAGGGTGCCAGTTTC-3′ and 5′-AATCCTTGGCCCTCTGAGAT-3′; *C/EBPα*, 5′-TTACAACAGGCCAGGTTTCC-3′ and 5′-GGCTGGCGACATACAGATCA-3′; *β-actin* (control), 5′-GACAACGGCTCCGGCATGTGCAAAG-3′ and 5′-TTCACGGTTGGCCTTAGGGTTCAG-3′. The amplification cycles included denaturation at 95 °C for 50 s, annealing at 55 °C for 1 min, and elongation at 72 °C for 50 s. After 30 cycles, the PCR products were separated by electrophoresis on a 1.5% agarose gel for 30 min at 100 V. The gels were stained with 1 mg/mL ethidium bromide and visualized with UV light using the BIO-RAD Gel Doc image analysis software (BIO-RAD Laboratories Inc., Irvine, CA, USA).

### 2.5. Western Blot Analysis

Western blotting was performed according to standard procedures. Briefly, cells were lysed in lysis buffer containing 50 mM Tris-HCl (pH 8.0), 0.4% Nonidet P-40, 120 mM NaCl, 1.5 mM MgCl_2_, 0.1% SDS, 2 mM phenylmethylsulfonyl fluoride, 80 μg/mL leupeptin, 3 mM NaF, and 1 mM DTT. Cell lysates were separated by 10% SDS-polyacrylamide gel electrophoresis, transferred onto a polyvinylidene fluoride membrane (Amersham Pharmacia, England, UK), blocked with 5% skim milk, and hybridized with primary antibodies. *PPARγ*, *C/EBPβ*, *C/EBPα*, *aP2*, *FAS*, Akt, phospho-Akt (*p*-Akt), *phsopho-GSK3β* (*p-GSK3β*), and *GSK3β* antibodies were from Cell Signaling and the monoclonal β-actin antibody was from Chemicon (Temecula, California, USA). HRP-labeled mouse anti-rabbit IgG were from Jackson ImmunoResearch (West Grove, PA, USA). The Chemiluminescence kit was from Pierce (Thermo Scientific, Rockford, IL, USA). After incubation with horseradish-peroxidase-conjugated secondary antibody at room temperature, immunoreactive proteins were detected using a chemiluminescent ECL assay kit (Amersham Pharmacia, UK) according to the manufacturer’s instructions.

### 2.6. Animal Experiments

The study protocol was approved by the Ethics committee for Animal Experiments of Gyeongsang National University. All rats were handled according to the University Guidelines and the Animal Care Committee Guidelines of Gyeongsang National University (GNU-181231-R0066). Forty male Sprague-Dawley (SD) rats weighing approximately 180 g were purchased from the Central Lab Animal Inc. (Seoul, Korea). Rats were acclimatized to the experimental facility for 1 week. All animals were kept under controlled temperature and on a 12 h light and 12 h dark cycle. The rats were divided into 3 groups of 10 and individually housed in polycarbonated cages in a room maintained at 22 °C and 55% relative humidity. All rats were allowed free access to their respective diets and drinking water for 5 weeks. Food intake was measured daily and body weight every two days. Obese rats were generated by feeding them a high fat diet (HFD). The normal diet group (ND, *n* = 10) was maintained on a normal diet based on a commercial diet (#55VXT0038, Samyang Co, Korea). The HFD group (HFD, *n* = 10) was fed an HFD based on a commercial diet (rodent diet with 60% kcal fat, Research Diet, Korea). The HFD plus ATM group (HFD + ATM, *n* = 10) was fed an HFD, and ATM (150 mg/kg body weight (BW)/day) or distilled water (ND group) was administered through the gastrointestinal tract.

### 2.7. Biochemical Assays

After 5 weeks on experimental diets, the rats were anesthetized, and the tissues were dissected and analyzed. The body weight and fatty tissue mass were measured with sensitivity limits of 0.1 g and 0.01 g, respectively. The body weight index was calculated by dividing the weight (g) by the square of body length (cm^2^). Blood was isolated from each rat, stored at 37 °C for 30 min, and centrifuged at 4000× *g* at 4 °C for 10 min to obtain plasma. The epididymal fat pad and perirenal fat pad were excised, weighed, and stored at −20 °C until assayed. The concentrations of plasma triglyceride (TG), total cholesterol (TC), and high-density lipoprotein (HDL)-cholesterol (HDL-C) were assayed enzymatically using commercial kits (Asan phams, Co., Seoul, Korea).

### 2.8. Histological Analysis

The epididymal fat pads and liver tissues were removed and fixed in 10% neutral buffered formalin. The fat pads and livers were subsequently embedded in paraffin, sectioned into 5 μm sections (Leica, Wetzlar, Germany), and stained with hematoxylin-eosin for microscopic assessment (Olympus, Tokyo, Japan). Three different cross-sectional areas and their cell populations were calculated using an image analysis program (Image-Pro Plus 6.0, Rockville, MD, USA).

### 2.9. Statistical Analysis

All experiment results were reported as the means ± SD and the experiments were repeated three–four times. The differences between groups were determined using ANOVA and Duncan’s multiple range tests. Values of *p* < 0.05 was usually considered as statistically significant.

## 3. Results

### 3.1. Effect of ATM on Intracellular Lipid Accumulation and TG Content in 3T3-L1 Cells

**Figure 1:**
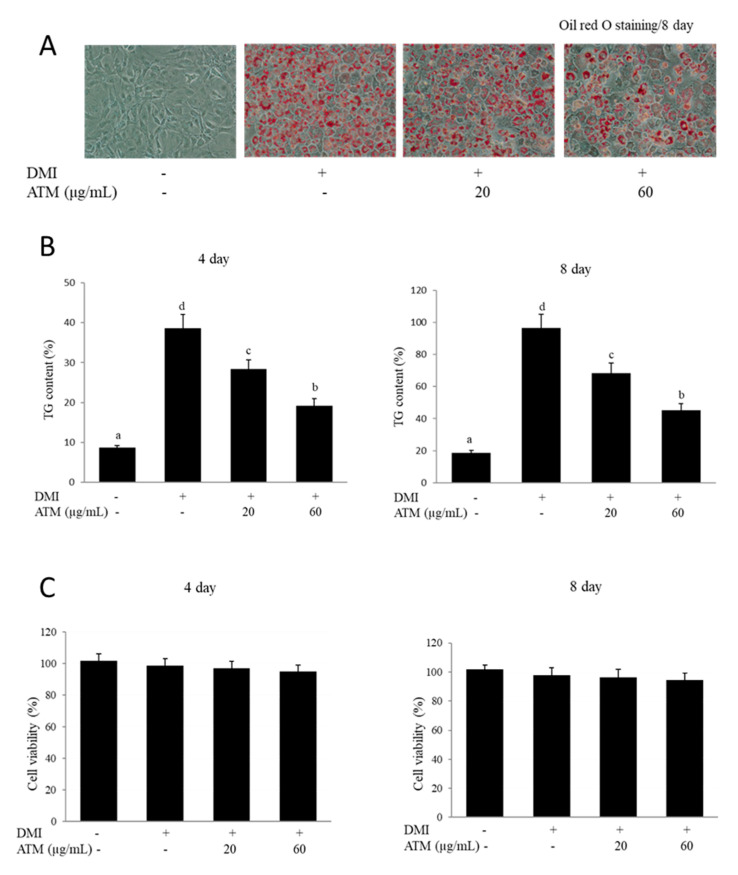
Effects of *Acer tegmentosum Maxim* (ATM) on lipid accumulation and adipocyte differentiation in *3T3-L1* cells. (**A**) *3T3-L1* preadipocytes were induced to differentiate in the presence or absence of ATM using MDI (0.5 mM 3–isobutyl–1–methylxanthine, 1 μM dexamethasone, and 0.5 μg/mL insulin). After 8 days of differentiation, cellular lipid content was assessed by Oil Red O staining. *3T3-L1* cells were induced to differentiate with desipramine (DMI) and ATM in increasing concentrations (0, 20, and 60 µg/mL) and lipid droplets on day 8 after differentiation were measured by Oil Red O staining. (**B**) Triglyceride (TG) content assay of *3T3-L1* cells on days 4 or 8 after the differentiation process. Intracellular TG contents were normalized by total protein. Three independent experiments were used to represent the error bars. The data shown are representative of at least three independent experiments. The values are presented as the means ± standard deviations (SD). Bars with different letters are significantly different (*p* < 0.05) as determined by Duncan’s multiple range test. (**C**) Effect of ATM on viability of *3T3-L1* cells was assessed by the MTT assay. The values are presented as the means ± SD. The data shown are representative of at least three independent experiments.

The anti-adipogenic effects caused by ATM on preadipocyte differentiation into adipocytes were investigated. The degree of adipogenesis of *3T3-L1* cells was measured by the amount of lipid accumulation using lipid-specific staining with Oil Red O at eight days after the induction of adipocyte differentiation. Differentiated adipocytes were identified and characterized based on the number of lipid droplets, which were not detected in undifferentiated cells. The quantification of Oil Red O staining demonstrated that treatment with 20 to 60 μg/mL of ATM significantly attenuated adipocyte differentiation. ATM strongly affected cellular lipid accumulation, which was decreased by approximately 40% in a dose-dependent manner (Figure 1A). Intracellular TG levels were also measured on day four or eight of *3T3-L1* adipogenic differentiation. The cellular TG content significantly increased in the DMI-treated group during *3T3-L1* differentiation for four or eight days, while the addition of ATM into the differentiation medium strongly blocked TG accumulation (Figure 1B). Together, the results from Oil Red O staining and TG content assay demonstrated that ATM caused suppression of *3T3-L1* preadipocyte differentiation and decreased adipogenesis, intracellular lipid accumulation, and TG contents.

### 3.2. Effect of ATM on Cell Viability

The effects of ATM on cell viability were measured with the 3–(4,5 dimethylthiazol–2, 5 diphenyl) tetrazolium bromide (MTT) assay. *3T3-L1* cells were exposed to various concentrations (20–60 μg/mL) of ATM extracts for four or eight days. Results from the MTT assay showed that 24 h incubation of cells with the ATM extract produced no toxic effects on cell viability at concentrations up to 60 µg/mL (Figure 1C). In this study, ATM concentrations in additional experiments were used up to 60 µg/mL.

### 3.3. Effect of ATM on the Expression of Adipogenic-Specific Genes during Adipocyte Differentiation

**Figure 2:**
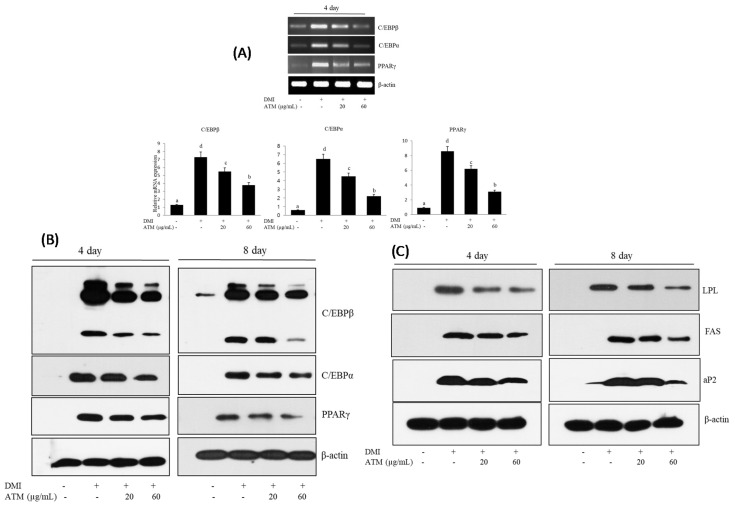
Effects of ATM on adipogenesis-related genes in *3T3-L1* adipocytes. (**A**) mRNA expression levels of *C/EBPβ*, *C/EBPα*, and *PPARγ* were measured in *3T3-L1* adipocytes on day 4 by reverse-transcriptase polymerase chain reaction (RT-PCR) analysis. Similar results were obtained by three independent experiments. Optical density analysis was performed to quantify the levels of mRNA expression with β-actin as the loading control. (**B**) The protein expression levels of *C/EBPβ*, *C/EBPα*, and *PPARγ* were measured in *3T3-L1* adipocytes by Western blot analysis. β-actin was used as the loading control. Bars with different letters are significantly different (*p* < 0.05) as determined by Duncan’s multiple range test. (**C**) Adipogenesis- and lipogenesis-related gene expressions in 3T3-L1 adipocytes on days 4 and 8 as determined by Western blot assay.

Adipogenesis was accompanied by an increase in adipogenic-related transcription factors and adipocyte-specific gene expression. In particular, *C/EBPβ*, *C/EBPα*, and *PPARγ* play a major role in adipogenesis. We examined whether ATM affected the mRNA levels related to expression of adopogenic transcription factors, such as *C/EBPβ*, *C/EBPα*, and *PPARγ,* using reverse-transcriptase polymerase chain reaction (RT-PCR) during adipocyte differentiation. As expected, the mRNA expression levels of *C/EBPβ*, *C/EBPα*, and *PPARγ* genes significantly increased in differentiated *3T3-L1* adipocytes (Figure 2A). ATM treatment caused significant down-regulation of *C/EBPβ*, *C/EBPα*, and *PPARγ* mRNA expressions in differentiated *3T3-L1* adipocytes.

Furthermore, the effects of ATM on the protein expression of the adipogenic transcription factors was examined using Western blotting analysis. C/EBPβ, *C/EBPα*, and *PPARγ* protein expression levels decreased in a dose-dependent manner in response to ATM in the 3T3-L1 adipocytes compared with those of untreated control cells (Figure 2B). Our data indicate that ATM induced inhibition of adipogenesis transcription factor expression with subsequent blocking of adipocyte differentiation.

### 3.4. Effect of ATM on the Expression of Lipogenesis-Specific Genes during Adipocyte Differentiation

Next, we investigated whether ATM could regulate the expression levels of lipogenesis-specific genes, such as *FAS*, *LPL*, and *aP2*. *C/EBPα* and *PPARγ* regulated the expression of their target genes, such as *FAS*, *LPL*, and *aP2*, during adipogenesis since those genes play a crucial role in determining and maintaining the mature adipocyte phenotype. Differentiated *3T3-L1* adipocytes showed an increase in *FAS*, *LPL*, and *aP2* expression levels, while treatment with ATM caused a significant, dose-dependent, down-regulation in the expression of these genes (Figure 2C). These results indicate that ATM produces strong inhibition of lipid accumulation and causes a reduction in lipogenesis-specific gene expression levels in *3T3-L1* adipocytes.

### 3.5. Effect of ATM on the Regulation of Akt and GSK3β during Adipocyte Differentiation

**Figure 3:**
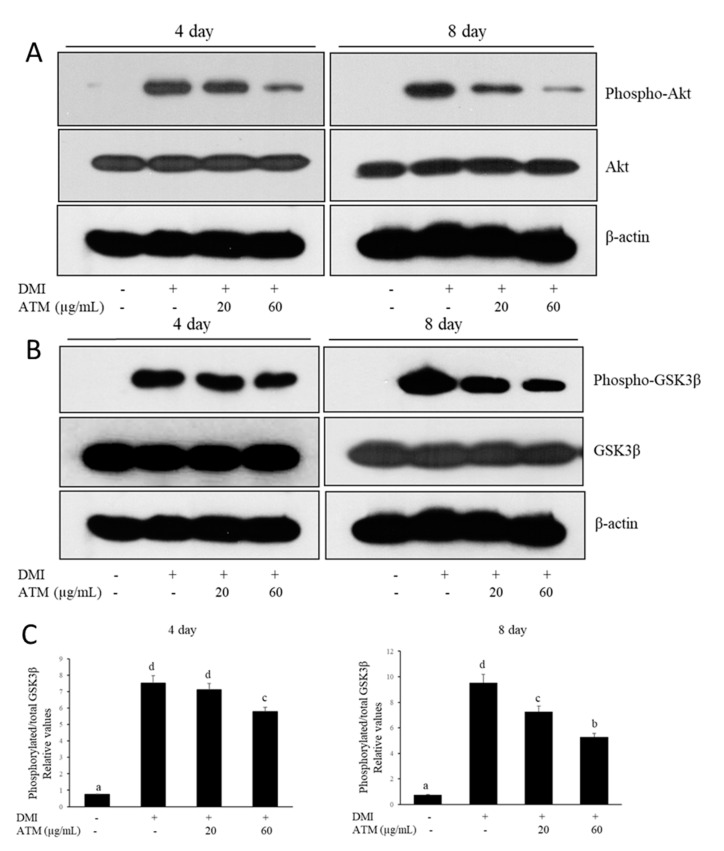
Effects of ATM on the Akt phosphorylation in *3T3-L1* adipocytes. (**A**) The phosphorylation of Akt in *3T3-L1* adipocytes. *3T3-L1* cells were differentiated with DMI in the absence or the presence of ATM for 4 or 8 days. The expression levels of Akt and phospo-Akt were measured in *3T3-L1* adipocytes by Western blot analysis. (**B**) Phosphorylation of GSK3β in *3T3-L1* adipocytes. *3T3-L1* cells were differentiated with DMI with or without ATM treatment for four or eight days. GSK3β and phospo-GSK3β expression levels were measured in *3T3-L1* adipocytes by Western blot analysis. (**C**) The phosphorylation of GSK3β was normalized to the total GSK3β expression level. Bars with different letters are significantly different (*p* < 0.05) as determined by Duncan’s multiple range test.

In order to investigate whether the Akt signaling pathway, a key regulator in adipogenesis, is activated by ATM during differentiation of *3T3-L1* cells, the phosphorylation level of Akt was examined after treatment with DMI alone or with DMI and ATM together. The results indicate that ATM treatment caused a significant and dose-dependent inhibition of phosphorylated Akt levels (Figure 3A). Moreover, ATM caused a reduction in the level of phosphorylated GSK3β, a downstream substrate of Akt (Figure 3B). These results demonstrate that activation of phosphorylated-Akt was inhibited by ATM; moreover, phosphorylation of GSK3β during adipogenesis in *3T3-L1* cells was also inhibited.

### 3.6. Effect of ATM on Body Weight and Adipose Tissue Weight in HFD-Induced Obese Rats

**Figure 4:**
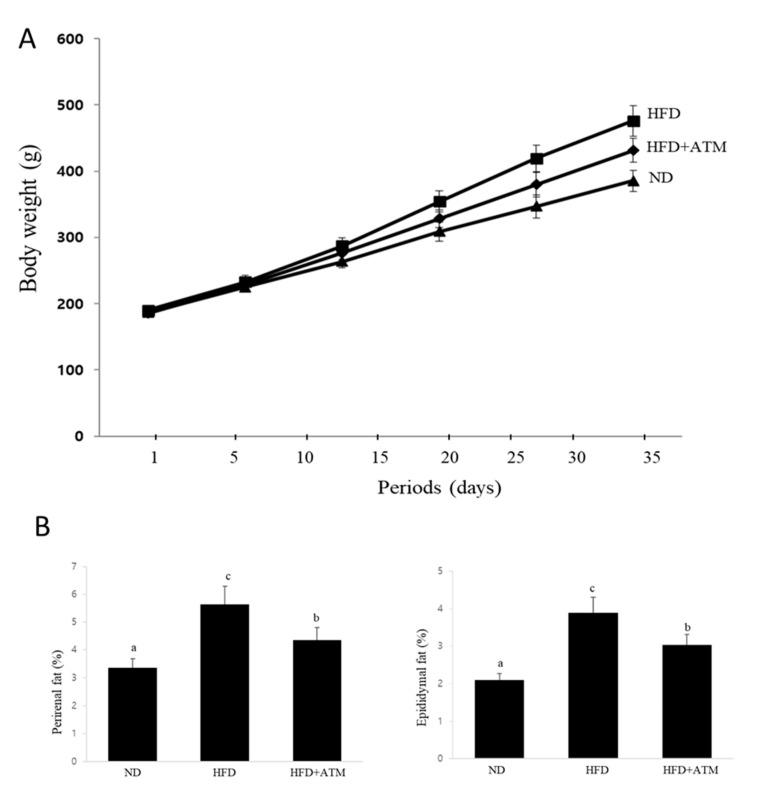
Effects of ATM on adiposity in HFD-induced obese rats. Rats (n = 10) were fed a normal diet or high-fat diet (HFD) for five weeks in the presence (150 mg/kg/day) or absence of ATM. (**A**) Effect of ATM on body weight in HFD-induced obesity in rats. The control group was fed a normal diet (ND), HFD + ATM groups were fed an HFD plus ATM (150 mg/kg body weight (BW)), and HFD groups were fed a high-fat diet (diet with 60% kcal fat). The BWs of each of the animals were measured twice a week. BWs at the end of the experiment were significantly different between the HFD and ND (*p* < 0.01) and HFD + ATM groups (*p* < 0.05). (**B**) Effects of ATM on perirenal and epididymal fat weights. The weights of perirenal and epididymal adipose tissue were measured by dividing fat tissue weight by body weight (fat tissue/body weight × 100%). The values are presented as means ± SD. Bars with different letters are significantly different (*p* < 0.05), as determined by Duncan’s multiple range test.

We further investigated the effects of ATM on body weight and adiposity in rats fed an HFD for five weeks. After one week of adaption, the rats used for this study were randomly distributed into three groups of ten rats: (1) a normal diet (ND) group; (2) high-fat diet (HFD) group; and (3) HFD group with ATM (150 mg/kg/day; HFD + ATM). Body weight (BW) at five weeks significantly increased in rats in the HFD group compared with that of the ND group, demonstrating that HFD-fed rats developed into obese rats (Figure 4A). Body weight gains were significantly lower in the HFD plus ATM group when compared with that of the HFD alone group. However, there were no significant differences in the amount of food intake in the HFD group compared with that of the other groups during the experimental period (data not shown). Thus, ATM-induced suppression of body weight gain did not depend on reduction of food and water intake. We also found that rats treated with ATM exhibited lower perirenal and epididymal fat mass compared to that of the HFD group (Figure 4B), demonstrating that the reduction in body weight gain was primarily due to reduced lipid accumulation in adipocytes.

### 3.7. Effect of ATM on Blood Biochemical Indexes in HFD-Induced Obese Rats

**Figure 5:**
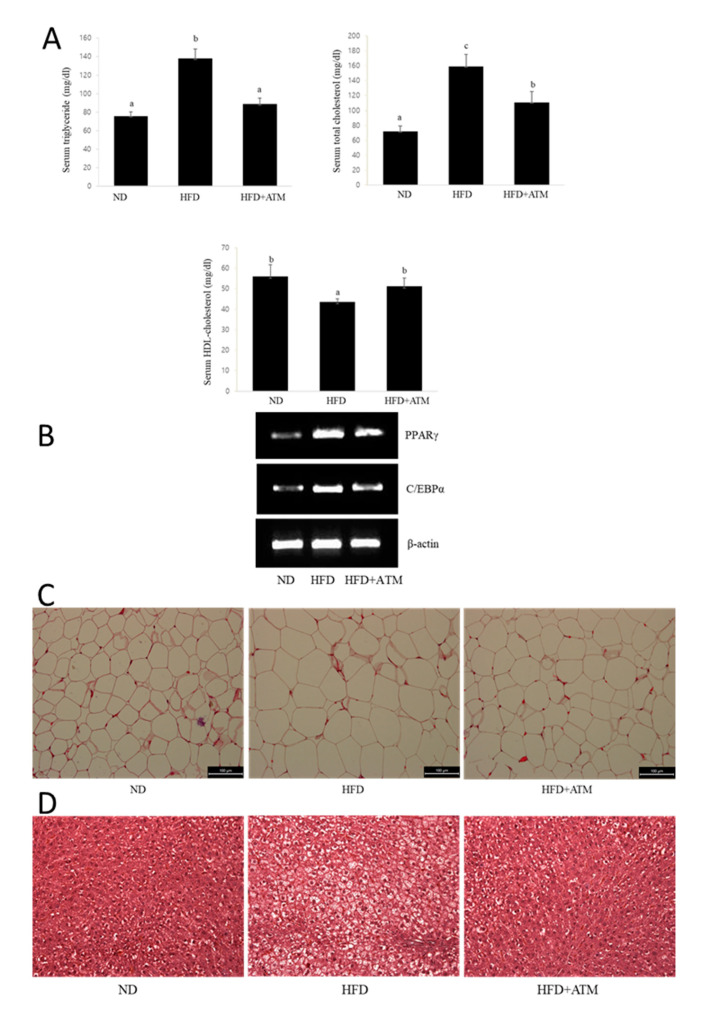
Lipid profiles and histological examination in rats fed with the HFD alone or HFD plus ATM. (**A**) Effects of ATM treatment on lipid content in HFD-induced obese rats. Significant decreases were observed in serum TG and total cholesterol (TC) levels of the ATM-treated group compared to the HFD alone group. High-density lipoprotein (HDL)-cholesterol (HDL-C) levels were significantly higher in the ATM-treated group compared to that in the HFD alone group. Values are presented as the means ± SD. Bars with different letters are significantly different (*p* < 0.05). (**B**) Effects of ATM on mRNA expression in epididymal adipose tissues. ATM administration depressed the expression of *C/EBPα* and *PPARγ* in the epididymal adipose tissue of HFD-induced obese rats. (**C**) Effects of ATM on histology of epididymal adipose tissue of rats fed an HFD. Histological analysis of epididymal adipose tissue after staining with hematoxylin and eosin (H&E) followed by microscopic analysis. Scale bar is 100 µm. (**D**) Effects of ATM on lipid accumulation in liver tissues. Histological analysis of liver after staining with H&E followed by microscopic analysis.

In order to further investigate the anti-obesity effects of ATM, we estimated the changes in serum TG, TC, and HDL-C levels in HFD-induced obese in rats. As shown in Figure 5, TG and TC levels were significantly higher in the HFD-fed rat group compared with those in the ND group (Figure 5A). However, TG and TC levels in the HFD group significantly decreased by 36% and 30%, respectively, following ATM supplementation. Moreover, serum HDL-C levels in the ATM group significantly increased compared to those in the HFD group (Figure 5A). Thus, the ATM extracts caused a significant decrease in serum TG and TC levels and increase in HDL-C levels.

### 3.8. Effect of ATM on Adipogenic Gene Expression or Lipid Accumulation in Epidydymal Adipose Tissue

We examined the mRNA expression of adipogenic genes in adipose tissue of HFD-induced obese rats. As expected, treatment of ATM reduced the mRNA expression of *PPARγ* and *C/EBPα* in epididymal adipose tissue. Next, in order to investigate whether the epididymal fat mass reduction in the ATM-treated group led to a decrease in epididymal adipose tissue cell size in obese rats, histological analyses of epididymal adipose tissue was performed. As shown in Figure 5, hematoxylin and eosin (H&E) staining results for epididymal tissue from each group showed that lipid accumulation significantly increased in the HDF compared with ND group (Figure 5B). However, the size of the adipocytes in the ATM administration group in the epididymal fat was significantly smaller than that in the HFD-fed rats (Figure 5B), and the size of adipocytes in the epididymal adipose tissue was reduced by 35% in ATM-treated rats.

### 3.9. Effect of ATM in Fatty Liver Tissue of HFD-Induced Obese Rats

Body weights of HFD-fed rats significantly increased in comparison to that of control rats after five weeks of feeding initiation. The HFD-fed rats exhibited obesity-related characteristics in terms of body weight, weight gain, and food intake compared to those in the ND group at the end of the study. H&E staining showed that the number of lipid droplets increased in the liver of rats undergoing HFD-induced obesity. Hepatic steatosis and ballooning degeneration of hepatocytes in the liver tissues were higher in the HFD than the ND group (Figure 5C). However, ATM administration significantly blocked the effects of HFD-induced hepatic steatosis (Figure 5C).

## 4. Discussion

Obesity is a medical condition in which abnormal or excessive fat accumulates mainly due to sedentary behavior that is linked to lower health-related quality of life, lack of exercise, and intake of foods rich in fats and oils. Trends in overweight prevalence have been increasing rapidly in children and adults and are associated with serious mortalities, including a high hyperlipidemia incidence, type 2 diabetes mellitus, insulin resistance, heart disease, and fatty liver in addition to various types of cancer and osteoarthritis [1]. The currently available treatment options are not potent enough to prevent obesity permanently, and other effective strategies for weight loss may cause other side effects. Therefore, there is a great demand for long-term use of complementary, alternative, and safe and effective medicines to treat this pandemic global obesity problem.

In the present study, we investigated the inhibitory effects of ATM on adipocyte differentiation in *3T3-L1* cell and the anti-obesity ATM activities on HFD-induced obese rats was investigated by analyzing body and fat pad weights, adipocyte size, and blood biochemical profiles.

ATM is well-known as a functional tree with potential medicinal benefits. Diarylheptanoids [24], rhododendrol glycoside [25], and tannins [26] have been isolated from the genus Acer. Other studies have also demonstrated that ATM is rich in abundant functional compounds, including polyphenols, phenethyl glycosides, and flavonoids, suggesting that these compounds exhibit hepatoprotective activities [20,21]. ATM exhibits potent antiangiogenic activity both in vivo and in vitro [27] and has been shown to be cytotoxic to cancer cells [21].

These results are the first to demonstrate that ATM causes inhibition of differentiation and adipogenesis of *3T3-L1* preadipocytes and lipid accumulation in mature differentiated adipocytes via regulation of adipogenic transcription factor expression and Akt signaling. In order to investigate whether ATM affects adipocyte differentiation, we measured the effects of ATM on lipid accumulation during *3T3-L1* preadipocyte differentiation. Adipogenesis is defined as the process by which preadipocytes differentiate into adipocytes. *3T3-L1* cells are induced to differentiate into adipocytes according to a coordinated program and are one of the most useful cell lines for studying adipogenesis [28]. In the present study, lipid accumulation was measured by Oil Red O staining and the TG assay. Our data demonstrated that ATM treatment caused a reduction in TG levels and a decrease in intracellular lipid content in a dose-dependent manner as quantified by Oil Red O staining in the cytoplasm of treated *3T3-L1* cells. These results demonstrate that ATM blocked adipogenesis during adipocyte differentiation via facilitating an effective decrease in lipid formation and lipid accumulation in *3T3-L1* adipocytes. To our knowledge, this is the first study showing the influence of ATM on lipid accumulation in *3T3-L1* adipocytes.

In line with these findings, we examined the effects of ATM on adipogenesis and lipid accumulation in *3T3-L1* adipocytes via regulation of adipogenic gene expression. Differentiation of preadipocytes is regulated by a complex network of transcription factors, mainly consisting of the *C/EBP* family and *PPARγ*. This cooperative function helps to maintain each of their own high expression levels. Moreover, adipocytes from mice in which the *C/EBPα* gene was disrupted showed defects in lipid accumulation [29]. Transgenic mice specifically lacking *PPARγ* in adipose tissue exhibited greatly reduced fat pad sizes [30]. *PPARγ* stimulates adipocytes and induces differentiation, while *C/EBPα* also supports the adipocyte differentiation process. Therefore, low levels of these adipocyte differentiation-specific genes are important markers of adipocyte inhibition.

In the present study, the treatment with ATM caused a decrease in mRNA expression of *C/EBPβ*, *C/EBPα*, and *PPARγ* in *3T3-L1* adipocytes. Western blot analysis showed that C/EBPβ, *C/EBPα*, and *PPARγ* protein levels during adipogenesis of *3T3-L1* preadipocytes demonstrated a dose-dependent reduction. These results show that the ATM treatment caused inhibition of adipocyte differentiation via inhibition of *PPARγ* and *C/EBP* family-associated adipogenesis signaling, and ATM effects on adipogenic transcription factors involved in adipocyte differentiation in *3T3-L1* cells play a critical role in adipogenesis mediation.

It is well known that adipocyte differentiation is associated with multifunctional cellular pathways and requires sequential regulation of adipogenic and lipogenic genes [31]. *PPARγ* and C/EBPα activate gene expressions, such as *aP2*, *LPL*, *FAS* and others, that are involved in adipogenesis and lipogenesis and participate in creating the adipocyte phenotype [8,10,11].

In the present study, the effects of ATM on the expression of *aP2*, *FAS*, and *LPL* were investigated during *3T3-L1* differentiation. The expression of *aP2*, *FAS,* and *LPL* genes is significantly down-regulated in a dose-dependent manner following ATM treatment. Together, our results demonstrate that ATM led to strong suppression of the expression of critical genes involved in creating and maintaining adipogenesis and lipogenesis via lipid storage and accumulation in *3T3-L1* adipocytes.

Several lines of evidence support the concept that the lipogenic pathway is localized to peroxisomes and is important for endogenous activation of *PPARγ*. *PPARγ* mainly regulates the gene network expression involved in adipogenesis and lipid metabolism [32]. *PPARγ* has a major role in the differentiation of preadipocytes into adipocytes involved in obesity development [33,34]. The fact that *PPARγ* null mice completely lack adipose tissue clearly demonstrates that *PPARγ* is essential for adipocyte differentiation [35]. Focusing on lipid metabolism genes, FAS regulates fatty acid synthesis from acetyl-and malonyl-CoA and via the tricarboxylic acid cycle [36]. Therefore, reduction of *FAS* via ATM treatment could explain the way in which ATM extracts suppress lipid accumulation. aP2 is highly expressed in adipose tissue and is a lipid binding protein, which reacts as a key factor in intracellular fatty acid transport and lipid metabolism [10,11]. The expression of lipoprotein lipase (LPL) is stimulated by *PPARγ*, thus increasing fatty acid uptake into adipocytes [11]. These genes are activated in response to *PPARγ* regulation [10,11]. In this study, ATM caused a reduction in the expressed level of *FAS*, *LPL*, and *aP2*, suggesting that intracellular fatty acid transport and lipid metabolism are reduced due to low lipid accumulation. The expression levels of those adipogenic and lipogenic-related genes decreased in ATM-treated *3T3-L1* adipocytes. These studies explain ATM-induced inhibition of adipocyte differentiation and lipogenesis in *3T3-L1* preadipocytes. Therefore, ATM reduced adipogenesis and lipid synthesis within differentiated adipocytes at the gene level through inhibition of the genes involved in adipocyte lipogenesis and fatty acid transport and synthesis, thus leading to lipogenesis.

Akt kinases play an important role in adipogenesis and glucose transport [37]. Inhibition of Akt activation in fibroblasts cause them to display a lack of capability to differentiate preadipocytes into adipocytes, and Akt overexpression results in an increase in glucose uptake and adipocyte differentiation [38]. In the present study, our results demonstrate that ATM caused a significant and concentration-dependent decrease in the phosphorylation of Akt in *3T3-L1* adipocytes. In addition, treatment with ATM caused a marked decreased in phosphorylation of GSK3β during adipocyte differentiation of the *3T3-L1* preadipocytes. It is known that Akt phosphorylation can promote adipocyte differentiation via *PPARγ* up-regulation [39]. The Akt signaling cascade is considered important for adipogenesis as it appears to activate *PPARγ* and *C/EBPα* during induction of *3T3-L1* adipocyte differentiation [39]. Xu et al. showed that Akt activation enhances an important association between the Akt signaling cascade and transcription factors, *PPARγ* and *C/EBPα*, in induction of *3T3-L1* adipocyte differentiation [17]. Taken together, these results demonstrate that ATM suppressed adipocyte differentiation and lipid accumulation in *3T3-L1* cells via down-regulation of adipogenic transcription factors and the Akt signaling pathway associated with intracellular lipid accumulation.

Rodent models of HFD-induced obesity have been widely used to investigate human obesity and its related metabolic diseases. Five weeks of an HFD leads to overt obesity in rats and is characterized by body weight gain along with an increase of fat tissue weight. In this animal study, we established that HFD-induced obesity in rats caused an increase in body weight of rats and serum TG and TC levels and a decrease in HDL-C levels. However, our results showed that BW gain was lower in rats administrated the ATM extract. The weights of epididymal and perirenal adipose tissue were also markedly reduced in rats supplemented with ATM, indicating that BW loss is mainly due to decreased fat accumulation in epididymal and perirenal adipose tissues. We also observed that serum TG and TC were lower in rats fed HFD plus ATM than those of rats fed HFD, indicating that ATM is beneficial for improvements in numerous serum metabolic parameters. Thus, these data suggest that ATM suppressed lipid disorders caused by the excess lipid found in HFD-induced obese rats, thereby improving hypertriglyceridemia and hypercholesterolemia caused by HFD-induced obesity. In addition, increased adipose tissue mass, particularly the amount of visceral fat, is associated with an increased risk of metabolic diseases. In this study, ATM-fed rats showed lower masses of epididymal and perirenal adipose tissues. Thus, supplementation with ATM caused reduction in BW and fat tissue weights, thus preventing fat accumulation and improving obesity.

Next, we investigated the effect of ATM extract for the prevention of fatty liver. The liver is mostly regarded as an essential organ in lipid metabolism. Imbalance between lipid deposition and removal results in hepatic lipid accumulation, which is related to an increase in hepatic lipogenesis, augmented lipid uptake, and/or reduced TG export of β-oxidation products [40]. In the present study, the liver tissue in the obese HFD-fed rats showed lipid droplet accumulation in hepatocytes, while the treatment with ATM caused a decrease in the deposits of hepatic lipids in liver tissue. These results indicate that the livers of ATM-treated rats had fewer adipocytes than that of the HFD-fed rats, thus inducing lipid droplet reduction in liver tissue. These findings highlight the critical role of ATM in reducing abnormal lipid accumulation in liver tissue in addition to causing a reduction in BW gain.

## 5. Conclusions

In this study, we showed the inhibitory effects of ATM on adipocyte differentiation. ATM treatment significantly inhibited lipid accumulation and adipocyte differentiation of *3T3-L1* preadipocytes in a dose-dependent manner. ATM caused a reduction in the expression of *C/EBPβ* and subsequent down-regulation of activation of the key transcriptional regulators, C/EBP family and *PPARγ*, in *3T3-L1* adipocytes. Furthermore, ATM caused a reduction in expression of adipogenesis-related factors, such as *FAS*, *aP2*, and *LPL*. We also demonstrated that ATM decreased lipid accumulation and adipogenic gene expression via inhibition of Akt signaling in *3T3-L1* preadipocytes that differentiate into adipocytes. Moreover, the administration of ATM effectively inhibited lipid accumulation in epididymal adipose tissue and liver tissue in HFD-induced obese rats. These results suggest that the anti-obesity effects of ATM result from a decrease in adipogenesis and that ATM has potential use as an anti-obesity agent.
